# A proposed mechanism for IS607-family serine transposases

**DOI:** 10.1186/1759-8753-4-24

**Published:** 2013-11-06

**Authors:** Martin R Boocock, Phoebe A Rice

**Affiliations:** 1 Institute of Molecular, Cell and Systems Biology, University of Glasgow, Scotland G12 8QQ, UK; 2 Department of Biochemistry and Molecular Biology, The University of Chicago, Chicago, IL 60637, USA

**Keywords:** IS607, Serine recombinase, Site-specific recombinase, Structure, Transposase, Transposition mechanisms

## Abstract

**Background:**

The transposases encoded by the IS607 family of mobile elements are unusual serine recombinases with an inverted domain order and minimal specificity for target DNA.

**Results:**

Structural genomics groups have determined three crystal structures of the catalytic domains of IS607 family transposases. The dimers formed by these catalytic domains are very different from those seen for other serine recombinases and include interactions that usually only occur upon formation of a synaptic tetramer.

**Conclusions:**

Based on these structures, we propose a model for how IS607-family transposases could form a synaptic tetramer. The model suggests that, unlike other serine recombinases, these enzymes carry out sequence-specific DNA binding and catalysis in trans: the DNA binding and catalytic domains of each subunit are proposed to interact with different DNA duplexes. The model also suggests an explanation for the minimal target DNA specificity.

## Background

**Figure 1:**
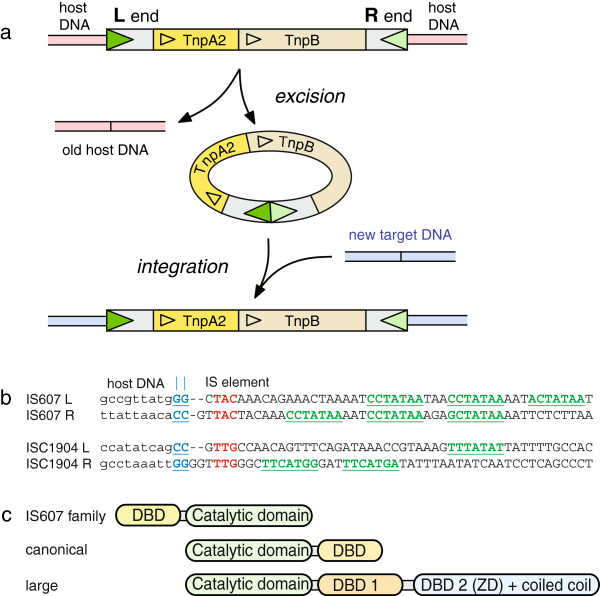
**Introductory cartoons. (a)** The organization of IS607 and its proposed transposition pathway. IS607 and related mobile DNA elements encode a serine transposase, TnpA2, and a second protein of unknown function, TnpB. The transposon ends are cartooned as green triangles. Excision creates a circular intermediate (NDF Grindley, personal communication) and reseals the original host DNA backbone. The circle can then integrate into a new location on the original DNA molecule, or, as cartooned, a new target DNA molecule (blue). **(b)** Sequences of the IS607 and ISC1904 ends. The deduced overlap dinucleotide at the host DNA/IS element junction (the crossover site) is in bold and underlined (blue); the small repeats that may be specific binding sites for the transposase are in bold (red), and other repeats are underlined (green). The latter repeats may be too far from the crossover dinucleotide for one protomer to interact with both motifs, and could play some other (regulatory?) role. The flanking host DNA sequences (lower case) are different at all insertion sites, and show little obvious pattern. **(c)** Domain organization of serine recombinases. The conserved catalytic domain of serine recombinases is always found at the N-terminus, except in serine transposases of the IS607 family, which have a MerR-family DNA binding domain at the N-terminus. “Canonical” serine recombinases, which include the resolvases and invertases, have a small C-terminal helix-turn-helix DNA binding domain. “Large” serine recombinases, which include bacteriophage integrases and some transposases, have a much larger C-terminal region that contains two DNA binding domains [12].

The IS607 family of insertion sequences (ISs) was first described in the human pathogens *M. tuberculosis* and *H. pylori*, and members of this family have now been identified in all three kingdoms of life and in eukaryotic viruses [1-6]. They usually encode two proteins: TnpA (sometimes called TnpA2), an unusual serine recombinase, and TnpB, a protein of completely unknown structure (Figure 1). Similar TnpB proteins are also encoded by IS200/IS605-type elements, but these encode a different TnpA that belongs to the Y1 (one tyrosine) family of transposases [7,8]. Although TnpB is conserved in both these IS families, it is not required for IS607 transposition, and it inhibits transposition of the IS200/IS605-family member ISDra2 [2,9]. TnpA is therefore the transposase of the IS607 family elements. Analysis of IS607 insertions in an *E. coli* system and genomic analysis of other family members showed that they insert with very little target sequence specificity [2,5]. This is very unusual for reactions catalysed by serine recombinases, which usually display extensive specificity for all recombining partners [10,11].

The serine transposases encoded by IS607-type insertion sequences represent a poorly understood branch of the serine recombinase family. Serine recombinases all share a common catalytic domain that includes the eponymous serine that is the active site nucleophile. The most intensively studied branch of this family, and the only one for which extensive structural information is available, comprises the “canonical” resolvase/invertase group [10]; these catalyse resolution of transposition cointegrates and replicon dimers, or inversion of DNA segments. A second branch that has also been biochemically characterized comprises the large serine recombinases, which include a number of bacteriophage integrases and some transposases. For both of the characterized groups, the catalytic domain is always found at the N-terminus of the protein and is followed by a sequence-specific DNA binding domain: a simple helix-turn-helix for the resolvase/invertase group, or a much larger bipartite domain in the large serine recombinases (hence their name) [12]. However, the IS607-family serine transposases carry a predicted helix-turn-helix DNA binding domain at the N-terminus, with the catalytic domain at the C-terminus (Figure 1c) [2,5,10].

**Figure 2:**
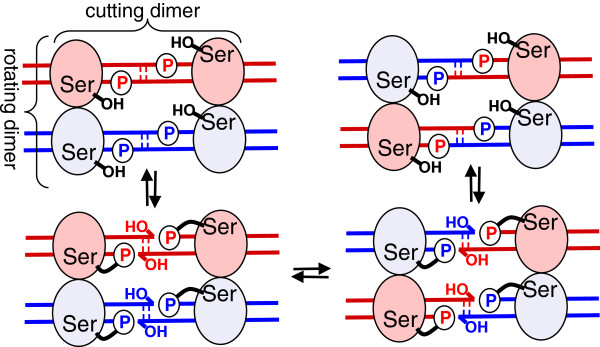
**Strand exchange by serine recombinases.** The cleavage, exchange, and religation of DNA strands occurs within a tetramer. Attack of the conserved serines displaces the 3′ hydroxyl groups, creating 2nt 3′ overhangs and 5′ phosphoserine protein-DNA linkages. Two subunits then rotate 180° to realign the broken ends, which are then religated to new partners by attack of the free 3′ hydroxyls on the phosphoserine linkages. A pair of subunits that binds and cuts the same initial duplex is termed a cutting dimer, and a pair that rotates together is termed a rotating dimer (see brackets in first panel). The full binding site for each cutting dimer, termed a “crossover site”, is an inverted repeat of two specific “half sites”, one on each side of the central dinucleotide.

Recombination by the characterized serine recombinases proceeds within a tetrameric complex that synapses the two DNA partners (Figure 2; reviewed in [10]). DNA is cleaved by attack of the conserved serine on a particular phosphodiester bond in the DNA, displacing a 3′ hydroxyl and creating a covalent protein-DNA intermediate. Once the DNA is cleaved, two subunits are thought to rotate 180° relative to the other two, carrying the broken DNA ends with them [13-19]. Religation then occurs by the chemical reversal of the cleavage reaction: the 3′ hydroxyls attack the phosphoserine linkages of their new partners. The recombination reaction is chemically reversible and these systems rely on additional features to determine when and in what direction it occurs. The tetramer can be subdivided into dimers in two different ways: “cutting dimers” in which both subunits bind (and cut/religate) the same duplex and “rotating dimers” in which the two subunits bind different duplexes but rotate together during the strand exchange process (Figure 2).

For the well-characterized serine recombinases, activation entails a pair of inactive cutting dimers undergoing large conformational changes as they come together to form a catalytically active tetramer. In the resolvase and invertase systems, activation is triggered by formation of a large synaptic complex that has a defined topology and includes additional copies of the recombinase and/or other DNA bending proteins [20,21]. This requirement can be bypassed by mutations that tip the conformational balance from the inactive dimer normally favoured by the isolated wild type (WT) protein to the catalytically active tetramer [10,22,23]. Integrases of the large serine recombinase family can form synaptic complexes without accessory factors, but usually only if they involve certain pairs of DNA sites (e.g., attP, and attB, the attachment sites found in the phage and bacterial DNA, respectively) [11].

**Figure 3:**
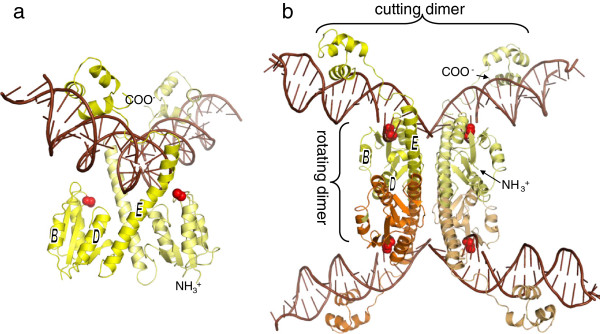
**γδ resolvase dimer and tetramer structures. (a)** Inactive dimer: WT γδ resolvase dimer bound to its cognate crossover site (PDBid 1gdt) [26]. Subunits are coloured yellow and pale yellow, with the side chains of the active site serines, which are distant from the DNA backbone, shown as red spheres. Helices B, D, and E are labelled for comparison with later figures. **(b)** Active tetramer: An activated γδ resolvase tetramer synapsing two crossover sites (PDBid 1zr4) [19]. The DNA is cleaved, with the 5′ ends covalently attached to the serines, and 2nt 3′ overhangs unpaired in the centre.

Structural studies have revealed similar cutting dimers formed by the WT catalytic domains of enzymes from both the canonical and the large serine recombinases, in which the active site is not fully assembled (e.g., Figure 3a) [24-26] and unpublished structures with protein database identifications (PDBids) 3g13, 1guv]. The one partial exception to this rule is the catalytic domain of TP901-1 integrase, which crystallized as a tetramer [27]. However, further biophysical data showed that it is dimeric in solution [27]. Tetrameric structures have also been determined for activated mutants of 3 canonical serine recombinases (Figure 3b) [19,28-30]. These show that the conformational changes that accompany activation create a remarkably flat central interface about which subunit rotation could occur. The conformational changes include disruption of the cutting dimer interface contacts, rotation and repositioning of the “core” of the catalytic domain relative to the last helix (“E”), and repacking of the 4 copies of helix E in the centre of the tetramer. Rotating dimer interactions are formed primarily by the antiparallel packing of the E helices of subunits bound to different duplexes.

It has been unclear how to apply the lessons learned from the well-characterized serine recombinases to the IS607-family serine transposases. For instance, in the canonical serine recombinases, the catalytic and DNA binding domains of each protomer interact with the same DNA half-site [26,31], but this is difficult to model for enzymes such as IS607 transposase where the DNA binding domain is N-terminal to the catalytic one, and attached by a rather short linker (~6aa for ISC1904). This geometry places the DNA binding domain (DBD) on the opposite side of the catalytic domain from the active site. Additionally, the current paradigm in which each of the four subunits binds one copy of a specific sequence motif (a “half site”) is hard to reconcile with the lack of target specificity shown by IS607-family transposases. Recently, structures of the catalytic domains of three different archaeal IS607-family serine transposases, including that from ISC1904 [32], have been determined by the Midwest Center for Structural Genomics (PDBids 3ilx, 3lhk and 3lhf). These reveal a very different dimer architecture than previously observed for other serine recombinases and suggest a different pathway for formation of an active tetramer.

## Results

### The IS607-family dimer is a “rotating” rather than a “cutting” pair

**Figure 4:**
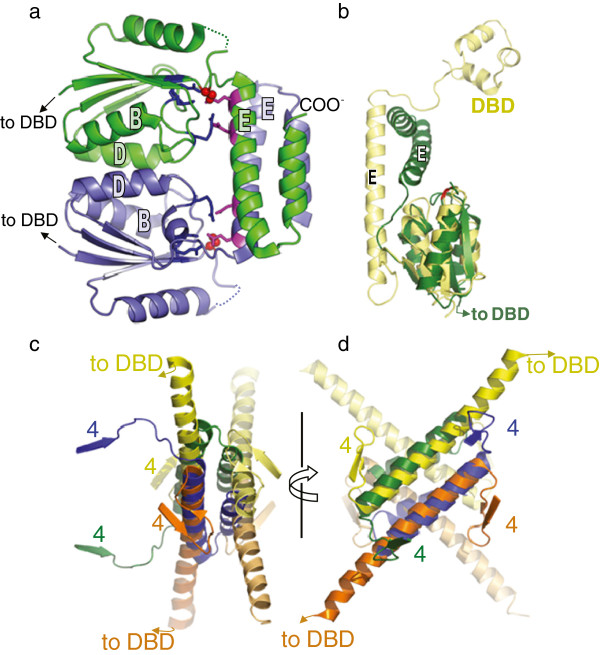
**Comparison of serine transposase dimer and resolvase structures. (a)** The dimer formed by the catalytic domains of OrfA from ISC1904 from *Sulfolobus solfataricus* P2 (PDBid 3ilx). The N-terminal DNA binding domain was removed before crystallization. One subunit is in green, one blue, the active site serines are shown as red spheres, three of the active site arginines are shown as blue sticks, and two negatively charged residues conserved only in IS607-family serine transposases are shown as magenta sticks. **(b)** Superposition of one subunit from the γδ resolvase dimer shown in Figure 3a (pale yellow) with one subunit of the serine transposase (green). The cores of the catalytic domains, comprising a 4-stranded beta sheet and the first 4 helices, have nearly identical tertiary structures. The structures diverge at a point identified as a flexible hinge in studies of other serine recombinases. **(c)** The serine transposase dimer’s E helices are packed similarly to those of the activated resolvase tetramer. The tetramer is shown in the same view and colouring as in Figure 3b, but only β strand 4 through helix E of each subunit is shown. The transposase dimer (blue and green) was superimposed on the left half of the tetramer using the E helices as guides. **(d)** Same as **(c)**, but rotated ~90° about a vertical axis.

The structure of the catalytic domain of ISC1904 transposase (from *Sulfolobus Solfataricus P2*; PDBid 3ilx) is shown in Figure 4 and is nearly identical to the dimers seen in the other two serine transposase catalytic domain structures. The packing of the E-helices in the serine transposase dimers more closely resembles that seen in activated tetramers rather than inactive dimers of canonical serine recombinases [19,25,26,28-30,33]. In contrast to other (inactive) serine recombinase dimer structures, the E helices pack against one another in an antiparallel manner and make sparse contacts with the rest of the catalytic domains. The C-terminal portions of the transposase E helices fold back on themselves at a point that is flexible in other structures [25,26]. The antiparallel packing of the E helices closely resembles that seen previously in activated tetramers between subunits that form rotating dimers (Figure 4c and d). Thus, we propose that the two subunits forming the dimer in the IS607-family transposases are those that will become a rotating dimer within an active tetramer. In contrast, for other serine recombinases the two subunits of the inactive dimer become a cutting dimer within the tetramer.

If the resolvase tetramer were to be split into two rotating dimers, a large hydrophobic surface would be exposed (Figure 3). In the serine transposase dimer, the equivalent surface is covered by the C-terminal portions of the E helices that fold back against it (Figure 4a). These observations suggested a model for the transposase in which a full tetramer is assembled on a single DNA crossover site. As described below, such a tetramer can be assembled by maintaining the packing between E helices within each dimer and by rotating about two flexible points within each subunit.

### Modelling an active IS607-family transposase tetramer

We anticipate that all IS607-family elements use a 'standard’ serine recombinase strand exchange mechanism (Figure 2; [10]) and transpose via a circular intermediate, similar to the circular forms of bacteriophages that use a large serine recombinase for integration/excision. In the circular form, the two ends of the mobile element would be joined to form a new crossover site through a specific 'overlap’ dinucleotide ('GG’ for IS607 [2]). Evidence for a circular form of IS607 was obtained by PCR in an *E. coli*-based transposition assay (NDF Grindley, personal communication). Recombination between the crossover site in the circular intermediate and a matching dinucleotide in the target DNA would insert the element into a new genomic location. Here, we propose a pathway for integration that can easily be extrapolated to the excision step.

To find a good model for the DBD, which was not included in any of the deposited serine transposase structures, we used the PROF routine of PredictProtein to predict its secondary structure [34]. This was consistent with a winged helix-turn-helix, with two short helices followed by a β-hairpin wing and a third short helix. The SoxR repressor begins with just such a DNA-binding motif and is also the top hit found by the Phyre2 threading server [35,36]. We therefore used a truncated version of the SoxR structure, with the DNA it was co-crystallized with, to model the DBD of the full-length ISC1904 transposase-DNA complex. The third helix of the transposase’s DBD is predicted to end at residue 47; this implies that there is a 6-residue linker before the catalytic domain, which becomes ordered in the crystal at residue 54.

**Figure 5:**
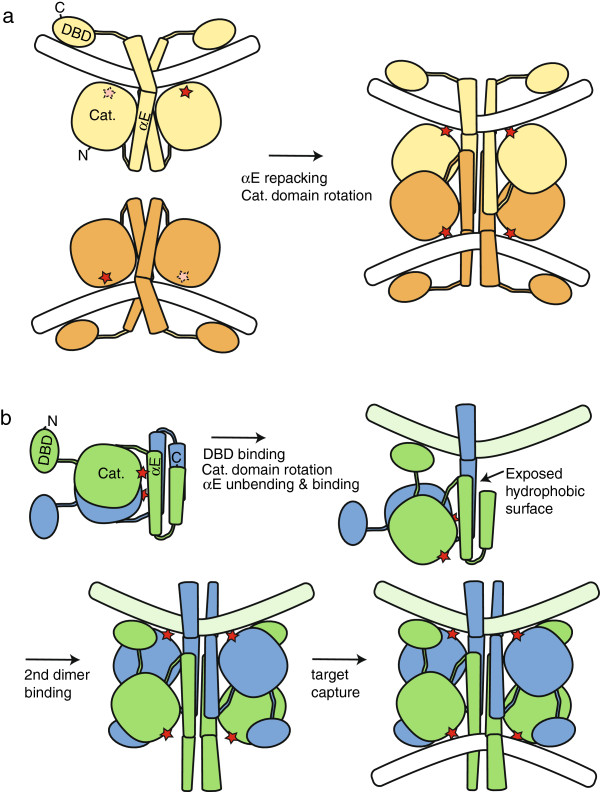
**Comparing the assembly of activated recombinase tetramers. (a)** Cartoons of the γδ resolvase dimer and tetramer structures shown in Figure 3, highlighting the conformational changes that occur during tetramer formation. Red stars denote active sites. **(b)** Proposed pathway for conversion of an IS607-family transposase dimer to an activated tetramer. The first panel (top left) cartoons the catalytic domain crystal structures, with a DNA binding domain added to the N-termini. In the second panel (top right), the green subunit’s DNA binding domain and the blue subunit’s E-helix bind the same DNA half site (DNA carrying specific binding sites for the transposase is green; this segment represents the junction of the left and right ends in the circular form, as cartooned in Figure 1a). Unbending of the E-helix so that its C-terminal segment can bind DNA exposes a hydrophobic surface, which is satisfied by interactions with a second dimer (third panel; bottom left). A tetramer is thus formed on one DNA duplex. The conformational changes required to form this tetramer prearrange the remaining DNA binding moieties (the blue DBDs and the C-termini of the green subunits), which would lower the energy barrier to their interacting non-specifically with a target DNA of near-random sequence.

**Figure 6:**
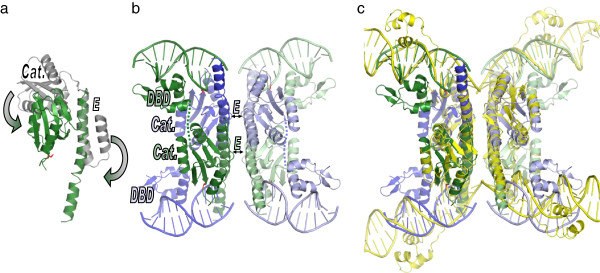
**IS607-family transposases and serine resolvases could form very similar activated tetramers. (a)** Diagram of the proposed conformation changes during activation of ISC1904 transposase. One subunit from the crystallized dimer (grey) is superimposed on one subunit from the modelled tetramer (green) via the N-terminal portion of helix E. Arrows show the proposed motions of the catalytic domain core and the C-terminal segment of helix E. These rearrangements do not alter the N-terminal portion of helix E, nor its interactions with its partner subunit within the dimer (not shown here). **(b)** Model of the ISC1904 transposase tetramer. The DNA binding domain and its interactions with DNA were modelled on SoxR, PDBid 2zhg [35]. The central two base pairs of each duplex were not modelled. **(c)** The ISC1904 model superimposed on the γδ resolvase tetramer (yellow; see Figure 3b) on which it was based. The misalignment of the lower right (light blue) DNA is due to a deviation of the resolvase structure from strict 222 symmetry.

The model for tetramer assembly (Figures 5 and 6), begins with the ISC1904 transposase dimer structure, with two SoxR-based DBDs attached by short flexible linkers to the N-termini. Straightening helix E of one subunit to more closely resemble known resolvase structures brings it into close proximity to the DBD of the opposite subunit. Since the C-terminal segment of helix E interacts with the minor groove of DNA in γδ and Sin resolvase-DNA structures, we predict that these structural motifs collaborate in binding a half-site (Figure 5b). The DNA was aligned with that in the γδ resolvase tetramer structure, although the central 2 bp, which are unpaired in the resolvase structure, were omitted from our model. The position of the DBD relative to the centre of the complex was based on the fact that the ISC1904 left and right ends both have a short sequence motif (TTG) that might comprise a specific binding site 2–4 bp from each 5′ end (Figure 1b; similar short repeats and conserved motifs can be found near the ends of many, but not all IS607-family elements listed in the ISFINDER database [2,5,6,37]). When the catalytic domain cores of each subunit are rotated about the β4-helix E hinge to match the position of those in the γδ resolvase tetramer, an interesting prediction appears: unlike well-characterized serine recombinases, this one will cleave the DNA in trans. That is, the DNA half-site that interacts with the green subunit’s DBD will become covalently linked to the blue subunit’s catalytic domain (Figure 5b).

Straightening the E helices of the dimer exposes a hydrophobic surface, which we propose interacts with that of a similar dimer (Figure 5b, third panel), triggering tetramer assembly. The order of the conformational changes proposed in Figure 5 is arbitrary. However, it is plausible that sequence-specific major groove binding by one subunit’s DBD would cause the C-terminal part of its partner’s E-helix to flip into the adjacent minor groove. Synergistic binding of a second dimer would result in a full tetramer bound to one DNA segment. Tetramer formation would force the other pair of E-helices (green in Figure 5b) into the extended conformation, ready to bind target DNA. Since the second set of DNA-binding moieties is thus pre-assembled, the tetramer’s affinity for target DNA of nearly-random sequence would be greatly increased over that of a single inactive transposase dimer. Note that all DNA binding proteins have some affinity for random-sequence DNA, although it can be orders of magnitude weaker than that for specific sequences. Our model implies that the serine transposase’s affinity for specific vs. non-specific DNA is tuned such that the affinity of a single DBD for non-specific DNA is too weak to be physiologically significant, but, due to cooperativity, a pre-assembled array of two DBDs plus two E helices binds non-specific target DNA tightly enough to be functionally relevant.

## Discussion

Figure 6 shows a ribbon drawing of the final model and a superposition of it onto the γδ resolvase tetramer structure. The catalytic domains and E-helices overlap quite well, with the only major difference lying in the placement of the DBDs. Note that the E-helix interactions of the initial dimer were maintained throughout the modelling. There is precedent for the type, if not the scale, of the inter-domain motions needed to construct the model. In several other structures, the E-helix bends and/or becomes disordered at the position where it folds back on itself in the IS607-family dimers [24-26] and PDBid 3 g13. Rotation of the catalytic domain core relative to the E-helix occurs in the transition from inactive dimer to active tetramer for both γδ and Sin resolvases and triggers assembly of the active site [19,28,29].

In the serine transposase case, an extra level of regulation may keep the dimer inactive until the proper complex is assembled: the active sites within the catalytic domain cores are physically occluded by the E-helices. The inhibitory interaction between the cores and E-helices is stabilized by two negatively charged side chains that interact with the conserved arginines of the active site (Figure 4a). This pair of negatively charged residues is highly conserved within the serine transposases but not within the larger serine recombinase family.

Another question is whether or not a tetramer assembled on one crossover site would repeatedly cleave and religate that site even in the absence of target DNA. Kinetic experiments suggested that Sin resolvase tetramers were catalytically active even when only bound to one DNA segment. However, those experiments bypassed the natural assembly pathway for Sin [38]. The serine transposases may have evolved an additional regulatory mechanism to avoid making double strand breaks until both DNA partners are present, which would be an interesting question to address experimentally. Preassembly of an active complex that captures a target site (of varying specificity) has good precedent in otherwise unrelated recombination systems, e.g., phage lambda integrase and the DDE family of transposases and retroviral integrases [39,40].

**Figure 7:**
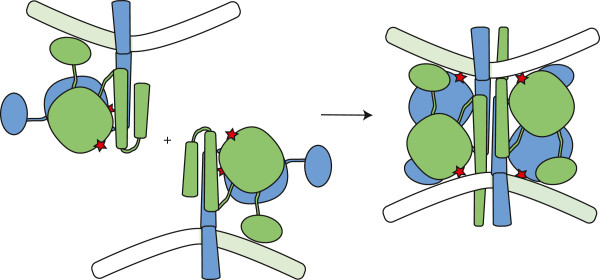
**Proposed assembly pathway for the excision reaction.** One rotating dimer could bind to each end of the element (specific binding sites in green), after which the two DNA-bound dimers would dock together to form an active tetramer. Note that the tetramers shown here and in Figure 5b can be interconverted by rotating their right halves by 180°.

The assembly pathway cartooned in Figure 5b regards the integration reaction, where one crossover site contains specific recombinase binding sites derived from the left and right ends of the IS element, and the other (“target DNA”) is non-specifically captured by a pre-assembled tetramer. How could this model accommodate the excision reaction, where only one arm of each crossover site contains any specific DNA sequences? Perhaps a tetramer could be nucleated by a single dimer binding to the sequence-specific half of each crossover site duplex. Unfolding of that dimer’s E-helices as they dock into the minor groove would expose a hydrophobic surface that would favour addition of the second dimer even though its DNA contacts would be non-specific. However, it is unclear how this assembly pathway would lead to proper synapsis of the two element ends and how recombination of one end with a random target would be avoided. Alternatively, the specific half-site at each IS end may bind one subunit of a rotating dimer and synapsis of the two ends may be mediated by two such dimers docking together (Figure 7).

## Conclusions

Our modelling exercise demonstrates that while the assembly pathway may be very different, the final activated tetramer formed by IS607-family serine transposases may be very similar to that formed by canonical serine recombinases. However, it would differ in that each subunit would act in trans – that is, the catalytic and DNA binding domains would interact with different DNA duplexes.

## Methods

Modelling was carried out by manipulating the relevant structures manually in Pymol (The PyMOL Molecular Graphics System, Version 1.3 Schrödinger, LLC).

## Abbreviations

DBD: DNA binding domain; IS: Insertion sequence; PDBid: Protein database identification; WT: Wild type.

## Competing interests

The authors have no competing interests.

## Authors’ contributions

MRB noticed that IS607-family transposase catalytic domain structures had appeared in the protein data bank and discussed the significance of their unusual dimer architecture with PAR. PAR constructed the model and wrote the manuscript, which MRB edited and approved. All authors read and approved the final manuscript.
